# Variances of quantifying of Virchow–Robin spaces detecting the different functional status of glymphatic system in simple febrile seizures affected by seizures duration

**DOI:** 10.1097/MD.0000000000032606

**Published:** 2022-12-30

**Authors:** Xin Li, Cailian Ruan, Yifan Wu, Mazen Musa, Abdoulaye Issotina Zibrila, Zhengxiang Zhang, Mustafa Salimeen

**Affiliations:** a Department of anesthesiology, School of Medicine, Yan’an University, Yanan,China; b Anatomy Department, School of Medicine, Yan’an University, Yanan City, China; c MD Undergraduate Program, School of Medicine, Yan’an University, Yan’an City, China; d Department of Orthodontics, Al Tegana Dental Teaching Hospital, Faculty of Dentistry, University of Science and Technology, Omdurman, Khartoum, Sudan; e Laboratory of Experimental Pharmacology, Department of Animal Physiology, Faculty of Science and Technology, University of Abomey-Calavi, Benin; f Department of Pharmacology, School of Medicine, Yan’an University, Yan’an City, China; g Department of Radiology, Affiliated Hospital, School of Medicine, Yan’an University, Yan’an City, China; h Department of Radiology, Dongola Teaching Hospital, Faculty of Medicine and Health Sciences, University of Dongola, Dongola City, Sudan.

**Keywords:** glymphatic system, magnetic resonance imaging, seizures duration, simple febrile seizure, Virchow-Robin spaces

## Abstract

The Virchow–Robin spaces (VRs) in the cerebral glymphatic system play a vital role in waste clearance from the brain. Simple febrile seizures (SFS) are a common type of seizures marked by an inappropriate fluid exchange. The mechanism of evident differences in glymphatic function among SFS with varying seizure duration is unknown. Therefore, the goal of this study was to see whether there were any variations in glymphatic function among SFS based on seizures duration. We retrospectively studied 30 children with SFS lasting more than 5 minutes (SFS > 5M), 40 children with SFS lasting 5 minutes or less (SFS ≤ 5M), and 35 healthy controls aged 6 to 60 months who underwent magnetic resonance imaging (MRI). A custom-designed automated method that used T2-weighted imaging (T2WI) to segment the visible VRs. The VRs metrics were measured and compared studied groups. The VRs metrics, seizure duration the time gap between seizure onset and MRI scan were studied as well. VRs counts were lower (*P* < .001) in the SFS ≤ 5M (445.80 ± 66.10) and the control (430.77 ± 182.55) groups in comparison to SFS > 5M (642.70 ± 100.62). Similar results were found for VRs volume (VRs_vol__SFS > 5M, 8514.63 ± 835.33mm^3^, VRs_vol__SFS ≤ 5M, 6390.43 ± 692.74 mm^3^, VRs_vol__control, 6048.37 ± 111.50 mm^3^; *P *< .001). However, in the SFS ≤ 5M, VRs measurements were lower than in the SFS > 5M (*P* < .001). VRs measurements were positively connected with seizure duration and inversely correlated with the course following seizure onset and MRI scan time in both SFS groups. SFS are positively correlated to glymphatic dysfunction since they cause enlarged VRs; additionally, VRs can be used as a biomarker in SFS > 5M and contribute to the mechanism.

## 1. Introduction

The cerebral glymphatic system, which is part of the brain’s immune system, is responsible for waste removal, leukocyte trafficking from the brain.^[1,2]^ Virchow-Robin spaces (VRs) in the brain parenchyma are functional networks of the cerebral glymphatic system that surround the perforating arterioles and venules.^[1,2]^ They’re always found between the endothelium basement membrane and the glia limitants’ basement membrane.^[3]^ Within the overall glymphatic system, the VRs are also in charge of immunological surveillance and the outflow of soluble proteins and metabolic waste products from the brain.^[4–6]^ The VRs and perivascular astrocytic aquaporin-4 (AQP-4) are involved in the interchange of cerebrospinal fluid (CSF) and interstitial fluid (ISF).^[1,7]^ Solutes and waste from the ISF enter the VRs, then travel to the cervical lymph nodes, where they are degraded, generating the brain glymphatic system circulation. These perivascular areas can become prominent and dilated on magnetic resonance imaging (MRI) if the circulation is compromised. As a result, VRs measures may reflect changes in glymphatic activity linked to neurological disorders.

The most prevalent neurologic diseases in children are seizures.^[8]^ Parents and family members experience anxiety relatively to seizure events in their children. Simple febrile seizures (SFS) are generalized seizures that last < 15 minutes and occur just once in 24 hours in a febrile child with no signs of intracranial infection, metabolic abnormality, or a history of afebrile seizures.^[9]^ SFS and complex FS are common seizure occurrences in children, affecting 2% to 5% of children aged 6 to 60 months.^[9,10]^ SFS is thought to make 70% to 75% of all FS.^[11]^ They are considered harmless and self-limiting, and they rarely result in long-term neurological problems.^[8]^ A prior study found no indication that SFS cause white matter (WM) alterations or developmental delays.^[12]^ However, children with SFS have a somewhat higher risk of developing epilepsy than others (1% vs 0.5%).^[10]^ Existing brain damage, a family history of seizures, or delivery difficulties could all play a role in the development of epilepsy or poor academic performance after FS.^[13]^ Even though SFS accounts for the majority of FS,^[11]^ the impact of SFS on glymphatic system alterations is unknown. Moreover, few studies have investigated whether SFS causes evident changes in glymphatic system dysfunction.

The mechanism underlying SFS is still unknown. Previous research has shown that the inflammatory process in the brain, particularly the release of cytokines, is linked to the development of FS.^[14]^ VRs and glymphatic system dysfunction are acute responses to SFS, according to a previous study.^[12]^ As a result, the inflammatory process may disrupt CSF-ISF exchanges and the blood-brain barrier (BBB).^[15]^ Visible enlarged VRs were observed on MRI in children with SFS lasting more than 5 minutes (SFS > 5M) and associated with seizure duration and course after the seizure onset (time interval between the seizure onset and MRI scan).^[16,17]^ This could be linked to aberrant CSF-ISF exchange and a leaky BBB in SFS > 5M.^[15,18]^ Consequently, we hypothesized that SFS > 5M could cause alterations in VRs, which could be linked to seizure duration and the time between seizure onset and MRI scans. However, no previous research on the relationship between VRs and SFS in children based on seizure duration has been conducted. Furthermore, it is unknown if VRs alterations show differences in seizure duration. Many methods for assessing VRs have been published, including visual rating scales developed by Banerjee et al,^[19]^ Potter et al^[20]^ and others,^[21]^ as well as quantitative methods based on custom-designed automated means.^[17,22,23]^ However, there are many discrepancies among visual rating methods for VRs, and sophisticated quantitative methodologies that allow an impartial assessment of VRs. Therefore, the goal of this study was to compare SFS > 5M, SFS of seizures duration of 5 minutes or less (SFS ≤ 5M), and controls using a custom-designed automated technique.

## 2. Materials and Methods

The local institutional review board, the Clinical Research Ethics Committee of Yan’an University’s Affiliated Hospital, examined and approved this retrospective study (No. YUSMAFEC2017RC-035). The children’s parents were aware of the potential risks of an MRI examination, such as loud noise. The parents had given written informed consent.

### 2.1. Participants

Two neuroradiologists with knowledge and experience of more than ten years interpreted the images and wrote the MRI reports for the participants. If there was a discrepancy between them, a third neuroradiologists with a combined experience of more than 20 years was consulted to help with the confirmed diagnosis. Children aged 6 to 60 months who completed MRI tests as part of the screening for brain disorders at the Department of Radiology in Yan’an University’s Affiliated Hospital were consecutively enrolled between May 2017 and December 2021. Children with SFS were included in this study if they met the following criteria: diagnosis of SFS based on the American Academy of Pediatrics’ criteria^[9,11]^; gestational age of 37 weeks or more; no history of brain injury, head trauma, or central nervous system infections; and (iv) a time interval of not more than15 days between seizure onset and MRI scan. The following were the exclusion criteria: insufficient clinical data on the course of the seizure after it initiated; insufficient clinical data on the duration of the seizure; and MRI abnormalities, such as hyperintensity on T2 fluid-attenuated inversion recovery (FLAIR) (except peritrigonal terminal zone of WM myelination that presents as a bilaterally symmetrical, slightly increased signal intensity with a hazy border on T2WI).^[24]^ The children in the control group met the following criteria: gestational age of fewer than 37 weeks; no history of SFS or other types of seizures; and no abnormalities on MRI. Children whose images revealed artifacts were not allowed to participate. Children with neurological problems (such as facial palsy and tic disorders) and intracranial infection were also excluded from the study.

### 2.2. MRI data acquisition

At the Department of Radiology, the Affiliated Hospital of Yan’an University, all participants underwent MRI examinations using the same 3.0-T scanner (Signa HDxt, GE Healthcare, Milwaukee, WI) with an 8-channel head coil. All children were adjusted while sleeping to eliminate motion artifacts and facilitate the MRI examination. Hearing protection was provided by micro-earplugs, while head immobilization was provided by molded foam. Fast spin-echo (FSE) T2-weighted imaging (T2WI), T2 FLAIR. Three-dimensional fast spoiled gradient-recalled echo T1-weighted imaging (T1WI), fast spin-echo (FSE), T2-weighted imaging (T2WI), T2 FLAIR. The parameters of MRI sequences (T1WI, T2WI, and T2-FLAIR) were the same for all participants. T2WI was utilized to assess VRs, and T1WI and T2-FLAIR were used to distinguish VRs from other WM hyperintensity lesions. The following were the parameters of the MRI sequences: T2WI: TR/TE = 4.200/120 ms; slice thickness = 4 mm without gap; FOV = 240 mm; and matrix size = 320 × 320.

### 2.3. VRs segmentation

To automate the segmentation of VRs in the WM above the bilateral ventricles (including the bilateral ventricular level), a custom script was built in MATLAB (R2012b; Math Works, Natick, MA).^[21,25]^ The skulls were eliminated during the segmentation process using ITK’s custom software (https://www.itk.org). The FMRIB software library (FSL) extracted the WM, gray matter, and cerebrospinal fluid areas from the images (https://fsl.fmrib.ox.ac.uk/fsl/fslwiki). The VRs were then quantified using images produced from FSL using a custom-designed algorithm in MATLAB. Two-dimensional (2D) Frangi filtering, which allows the filtering of vessel-like tubular structures from 2D images of axial plane T2 images, was at the core of the algorithm used to segment visible VRs.^[25]^ The segmentation yielded the brain volume, WM volume, VRs count, and VRs volume.

### 2.4. Statistical analysis

Locally weighted smoothing (LOESS) was conducted to detect the cutoff points of seizures duration in SFS, then the participants were divided in to 2 groups based on seizures duration in to SFS > 5M and SFS ≤ 5M. To compare demographic and clinical data between the SFS > 5M, SFS ≤ 5M, and control groups, the Mann–Whitney U test was performed. The Mann–Whitney U test was used to assess the differences in VRs counts and volume across the 3 groups. Using Pearson correlations, the connections between VRs counts and volume and seizure duration and course after initiation were investigated. IBM’s SPSS software (Version 21.0; Armonk, New York) was utilized for statistical analysis. *P* < .017 was determined to be statistically significant using the Bonferroni correction, and multiple comparisons were made across the 3 groups.

## 3. Results

**Figure 1.**
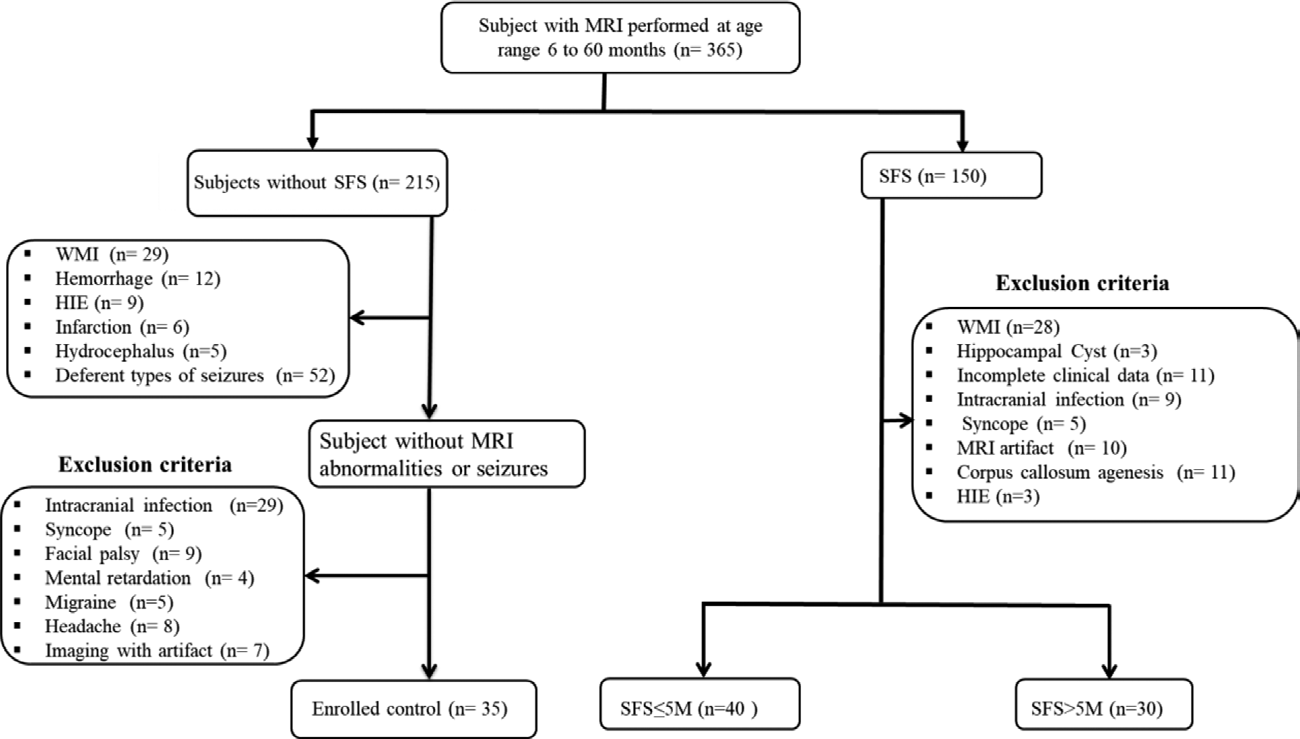
Participants flow chart for assuming research based on the inclusion and exclusion criteria. WMI = white matter injury, HIE = hypoxic-ischemic encephalopathy.

**Figure 2.**
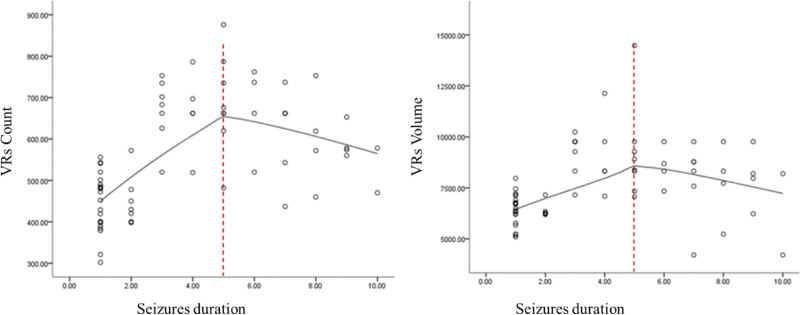
LOESS showed 5 minutes is the cutoff point for VRs to involve in the mechanism of SFS. SFS = simple febrile seizures, VRs = virchow–robin spaces.

This population-based study eventually enrolled 105 children based on the inclusion and exclusion criteria: 30 in the SFS > 5M, 40 in the SFS ≤ 5M, and 35 in the control group (Fig. 1). Among SFS groups 5 minutes was used as the cutoff point between them based on LOESS findings (Fig. 2). There were no significant differences in age, sex ratio, gestational age, or scan of duration after seizures onset between the SFS > 5M, SFS ≤ 5M, and control groups in terms of age, sex, gestational age, or scan of duration after seizures onset (Table 1).

**Table 1:** Participant’s demographics and clinical data.

	SFS > 5M (n = 30)	SFS ≤ 5M (n = 40)	Control (n = 35)	*P* value		
				SFS > 5M vs SFS ≤ 5M	SFS > 5M vs Control	SFS ≤ 5M vs Control
Age (mo)	28.07 ± 15.12	26.89 ± 15.92	27.83 ± 17.04	.686	.791	.875
Gender(male)	28(70 %)	22(73.33 %)	26(74.29 %)	.762	.682	.931
GA (w)	39.73 ± 1.07	39.80 ± 0.94	39.41 ± 1.14	.830	.189	.137
Course duration after seizures onset (d)	9.60 ± 1.32	9.40 ± 1.43	NA	.662	NA	NA
Seizure duration (min)	7.16 ± 1.23	2.70 ± 0.45	NA	<.001**	NA	NA

SFS = simple febrile seizures, NA = Not applicable.

** *P *< .001. Note: data are presented as mean ± standard deviation.

### 3.1. Comparisons of VRs findings among children with SFS > 5M, SFS ≤ 5M, and controls

**Figure 3.**
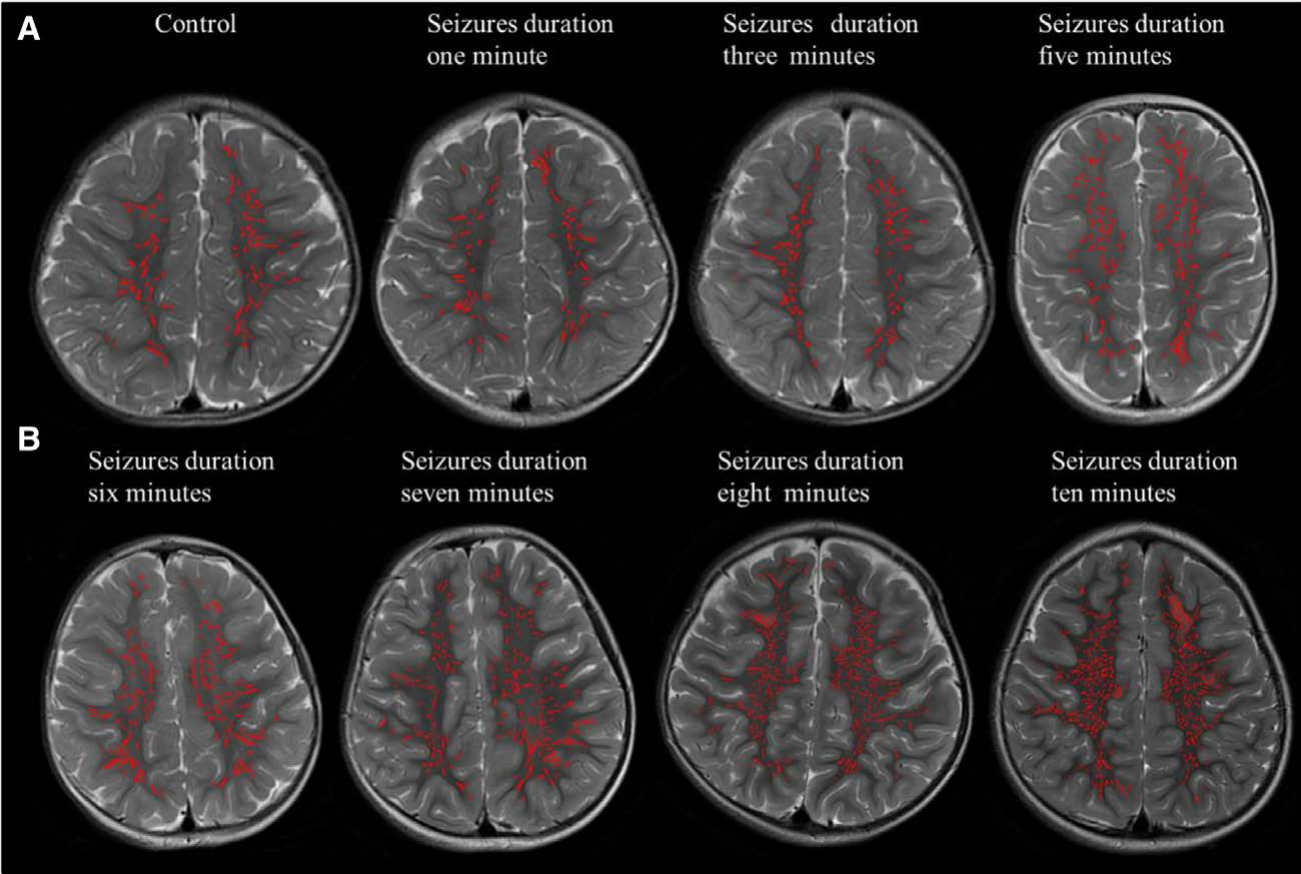
Automatic segmentation of visible Virchow-Robin spaces (VRs) at the supraventricular level in representative images. (A) VRs segmentation images in the control and SFS ≤ 5M. (B) Automatic segmentation images of VRs in the SFS > 5M. The circular and linear visible VRs were marked by red regions. SFS = simple febrile seizures, VRs = virchow–robin spaces.

Children with SFS > 5M, children with SFS ≤ 5M, and the controls group had their VRs counts, and volume assessed using an automated segmentation method (Fig. 2). There were significant differences in VRs counts and volume in SFS > 5M compared to SFS ≤ 5M and control groups (*P* < .001). The SFS > 5M had significantly higher counts and volume of visible VRs than the SFS ≤ 5M and control groups (*P* < .001). However, in the SFS ≤ 5M, visual VRs counts and volume were lower than in the SFS > 5M (*P* < .001). The volumes of the brain, WM and head circumference were not substantially different across the 3 groups (*P* > .017) as shown in (Fig. 3) and (Table 2).

**Table 2:** Compare VRs metrics among the SFS > 5M, SFS ≤ 5M, and control groups.

	SFS > 5M (n = 30)	SFS ≤ 5M (n = 40)	Control (n = 35)	*P* value		
				SFS > 5M vs SFS ≤ 5M	SFS > 5M vs Control	SFS ≤ 5M vs Control
VRs counts	642.70 ± 100.62	445.80 ± 66.10	430.77 ± 182.55	<.001**	<.001**	.62
VRs volume (mm^3^)	8514.63 ± 835.33	6390.43 ± 692.74	6048.37 ± 111.50	<.001**	<.001**	.35
WMV(×10^3^mm^3^)	300.67 ± 155.80	321.42 ± 70.52	308.51 ± 134.45	.64	.89	.83
HC (cm)	70.82 ± 10.18	71.64 ± 11.35	71.26 ± 10.04	. 69	.90	.90
BV(×10^3^mm^3^)	880.01 ± 156.41	894.29 ± 81.10	916.73 ± 181.91	.28	.13	.58

BV = brain volume, HC = head circumference, SFS = simple febrile seizures, VRs = virchow–robin spaces, WMV = white matter volume.

**Figure 4.**
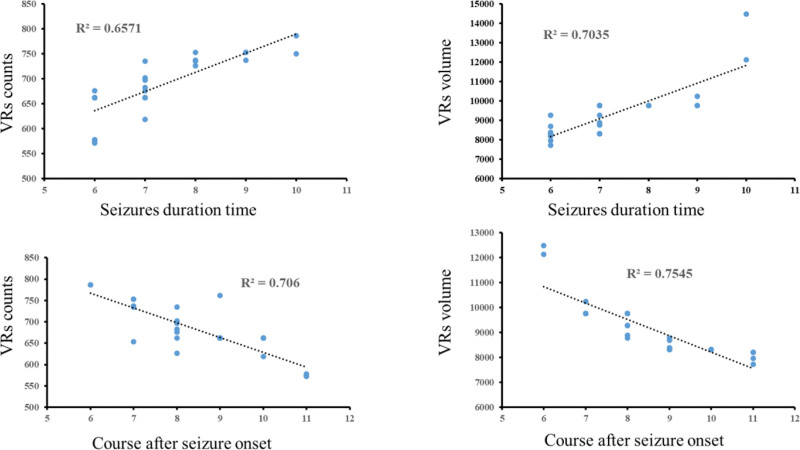
Correlations of VRs metrics and seizure duration time, and course after seizure onset in the s SFS > 5M (*P* < .001). SFS = simple febrile seizures, VRs = virchow–robin spaces.

**Figure 5.**
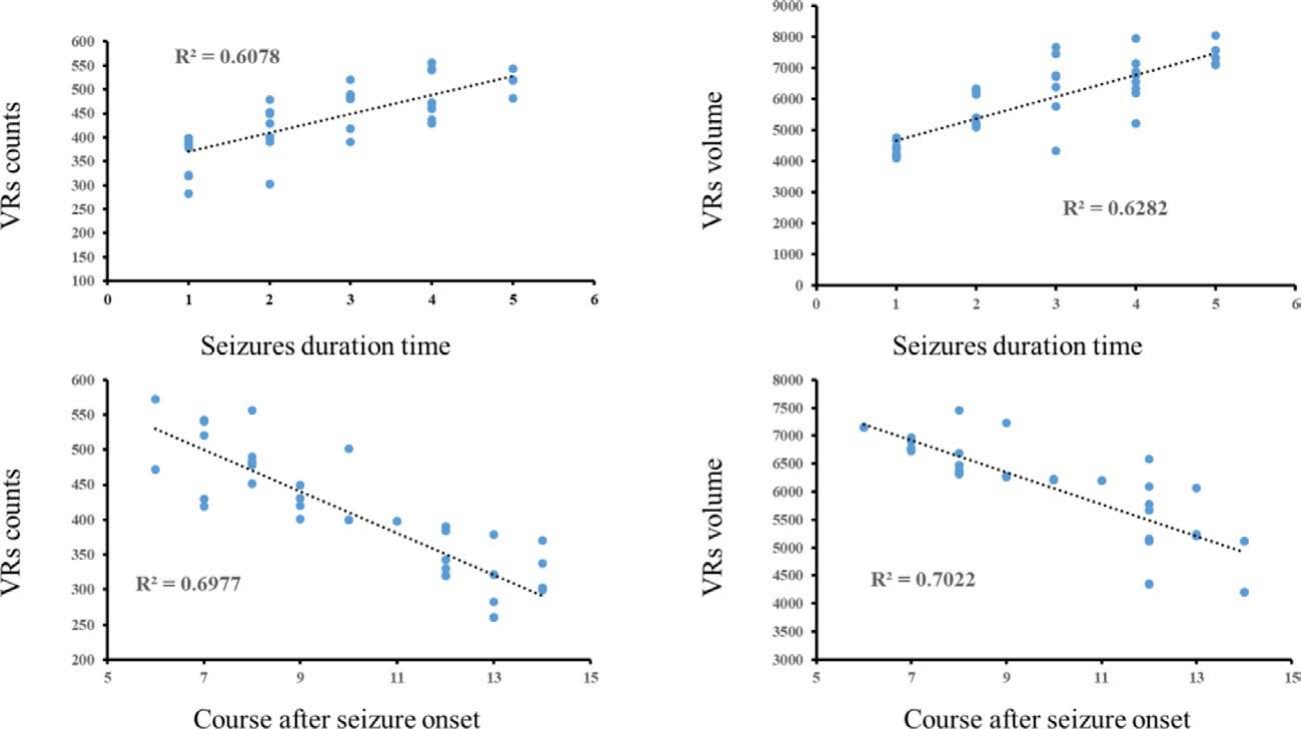
Correlations of VRs metrics and seizure duration time, and course after seizure onset in the SFS ≤ 5M group (*P* < .001). SFS = simple febrile seizures, VRs = virchow–robin spaces.

When comparing variations between VRs metrics and seizure duration, and the course after seizure onset in the SFS > 5M and SFS ≤ 5M groups, positive correlations were noticed between seizure duration and the VRs counts, and VRs volume in both SFS > 5M and SFS ≤ 5M groups (SFS > 5M: r__vol_ = 0.702, r__count_ = 0.778, *P* < .001; SFS ≤ 5M: r__vol_ = 0.568, r__count_ = 0.563, *P* < .001). In contrast, the course after seizure onset showed negative correlations with both VRs counts and VRs volume in both groups (SFS > 5M: r__vol_ = −0.807, r__count_=−0.812, *P* < .001; SFS ≤ 5M: r__vol_ =−0.562, r__count_ =−0.628, *P* < .001) (Figs. 4, 5)

## 4. Discussion

We assessed visible VRs counts and volume in the SFS > 5M and the SFS ≤ 5M. We also investigated the correlations between seizure duration, course after seizure initiation, and VRs metrics in this study. The VRs measurements were increased in the SFS > 5M than in the SFS ≤ 5M than and controls. Simultaneously, there was no statistically significant difference in VRs metrics between SFS ≤ 5M than and controls groups. In both the SFS > 5M and the SFS ≤ 5M there were positive correlations of VRs metrics with seizure duration and negative correlations of VRs metrics with the course after seizure onset.

The cerebral glymphatic system, specifically the VRs, is critical for CSF-ISF circulation and waste removal from brain parenchyma.^[1]^ Previous studies revealed that dilated VRs were linked to SFS and epileptic seizures.^[12,26]^ We used a custom-designed automated approach to quantify the VRs in controls and children with SFS > 5M and SFS ≤ 5M, utilizing the same MRI processing method as in previous studies.^[12,26]^ Our findings revealed that the VRs counts and volume were higher in the SFS > 5M than in the SFS ≤ 5M and control groups, which was consistent with prior findings^[12]^ that demonstrated, higher VRs metrics in SFS compared to control. Increased visible VRs have been linked to seizures and cerebral glymphatic circulation in children with SFS > 5M. The inflammatory process is a significant feature of seizures and SFS > 5M,^[18,27–29]^ despite the fact that the mechanism behind FS is unknown. By influencing the tight endothelial junctions and the basal membrane, proinflammatory mediators can cause BBB degradation.^[18,30]^ Through the compromised BBB, infiltrating leukocytes and albumin would accumulate in the VRs, causing dilatation of the VRs that are visible on MRI. BBB disruption may also play a role in aberrant CSF-ISF circulation.^[15]^ The VRs are involved in the CSF-ISF exchange, which is part of the glymphatic system circulation.^[1]^ The disruption of CSF-ISF exchange can obstruct waste and secretory product clearance, exacerbating VRs dilation.^[31]^ Furthermore, an accumulation of products within the VRs, such as albumin, and disordered ionic gradients obstruct waste removal and perivascular fluid flow, aggravating VRs dilation.^[15]^ Moreover, tau protein has been linked to axonal damage in the recurrent seizures brain.^[31,32]^ The elimination of tau protein by the glymphatic system was similar to that of β amyloid.^[33]^ However, animal models suggest that overexpressed tau protein will accumulate in the extracellular and perivascular regions due to inadequate clearance. As a result, increasing tau protein accumulation could obstruct the clearance of waste components from the brain, potentially compromising the VRs’ structural integrity.^[31,32,34]^ In children with SFS > 5M, our study found an increase in VRs counts and volume, which is consistent with the previous findings.^[12,26]^ Water channels like aquaporin 4 (AQP-4) promote convective flow out of the para-arterial region and into the interstitial space by facilitating glymphatic system function.^[1,35]^ When AQP-4 is mislocated to the cell body of astrocytes or to astrocytic processes that do not adjoin the vasculature, clearance of soluble proteins through the glymphatic system is significantly reduced.^[35]^ We believe that after seizure activity, AQP-4 may not operate properly, resulting in reduced metabolic product clearance and buildup in the VRs and interstitial space. According to our findings, the above-mentioned mechanisms cause an increase in metabolic product buildup during and after seizures, which could lead to an increase in visible VRs counts and volume. As a result, quantifying VRs parameters may aid in the investigation of links between glymphatic function and illnesses.

Nonetheless, the VRs counts and volume were lower in children with SFS ≤ 5M than in children with SFS > 5M.FS are a type of mild seizure that lasts < 15 minutes and usually does not recur within 24 hours or during the same illness.^[10]^ The previous studies revealed that seizure severity was associated with glymphatic system malfunction.^[12,26]^ VRs measures were also found to be positively correlated with seizure duration and negatively correlated with the course after seizure onset in our research. As a result, SFS may lead to a higher number of VRs counts and volume when seizures lasted longer than a couple of minutes. Because of our research, we established that seizure duration can cause glymphatic system dysfunction and that the severity of this dysfunction is connected to seizure severity.

With longer seizures, it was possible that more products would be released, resulting in more severe BBB failure and apparent VRs. In addition, vascular pulsation or hydrostatic pressure drive ISF exchange along the VRs.^[36]^ Sustained hypertension after seizure onset reduced arterial pulsation, decreasing metabolic clearance efficiency by the VRs,^[37]^ modifying neurovascular coupling, and increased BBB leakage by vascular oxidative stress.^[34]^ We anticipated that VRs function was affected and that the balance between increased metabolic product secretion and decreased clearance ability during the ictal phase of SFS was disrupted, based on our findings. Consequently, visible and dilated VRs (counts and volume) were higher in children with SFS of SFS > 5M groups with SFS ≤ 5M and controls. We also found that as the time delay between MRI and seizure start was extended, VRs counts and volume gradually reduced. Previous research has shown that BBB failure can be observed within minutes and within the first few days following a seizure,^[38]^ and that BBB damage caused by seizures can be reversed.^[15]^ Our findings suggested that as time passed after the onset of seizures, VRs function improved. The quantification of VRs characteristics may represent changes in the glymphatic system’s functional condition. Furthermore, in SFS, an increase in perivascular space was not linked to white matter injury in a prior investigation.^[12]^ Given that SFS involves relatively less severe seizures, we anticipate that changes in VRs metrics in SFS after 5 minutes of seizure duration could be used as a diagnostic biomarker for the disease’s pathophysiology. Seizures involving more than 5 minutes may be accompanied by glymphatic dysfunction, which could be a prelude to seizure-induced brain injury. However, better research designs are required to demonstrate the link between glymphatic system malfunction and SFS.

There were a few limitations in our research. First, this was a retrospective cross-sectional study with limited sample size, and these children were not fellow up. A follow-up study is presently underway to understand the role of the quantified VRs in glymphatic function alterations. Second, in patients with SFS groups, clinical data such as cytokine levels were not examined. These findings, on the other hand, may strengthen the link between seizure duration and glymphatic function. In the future, a prospective study will be needed to evaluate the relevance of quantified VRs in SFS > 5M as a biomarker, allowing clinicians to track glymphatic function disease development and choose the best treatment approach.

## 5. Conclusion

SFS > 5M can induce alteration in VRs counts and volume. Quantitative measurements of VRs detectable on MRI in SFS are linked to glymphatic system malfunction and may be used to identify potential biomarkers involved in the mechanism. The duration of the seizure and the course following the seizure onset are connected to the changes in these parameters in VRs.

## Acknowledgments

We appreciate the contribution of our children and their parents for participating in current study.

## Author contributions

**Conceptualization:** Xin Li.

**Software:** Abdoulaye Issotina Zibrila.

**Supervision:** Mustafa Salimeen.

**Validation:** Yifan Wu, Mazen Musa, Mustafa Salimeen.

**Writing – review & editing:** Cailian Ruan, Zhengxiang Zhang.

## References

[1] Plog BA Nedergaard M. The glymphatic system in central nervous system health and disease: past, present, and future. Annu Rev Pathol Mech Dis. 2018;13:379051217–394. 10.1146/annurev-pathol-051217-111018 PMC5803388 29195051

[2] Abbott NJ Pizzo ME Preston JE. The role of brain barriers in fluid movement in the CNS: is there a “glymphatic” system? Acta Neuropathol. 2018;135:387–407. 29428972 10.1007/s00401-018-1812-4

[3] Zlokovic BV. The blood-brain barrier in health and chronic neurodegenerative disorders. Neuron. 2008;57:178–201. 18215617 10.1016/j.neuron.2008.01.003

[4] Ramirez J Berezuk C McNeely AA. Imaging the perivascular space as a potential biomarker of neurovascular and neurodegenerative diseases. Cell Mol Neurobiol. 2016;36:289–99. 26993511 10.1007/s10571-016-0343-6 PMC11482437

[5] Jessen NA Munk ASF Lundgaard I. The glymphatic system: a beginner’s guide. Neurochem Res. 2015;40:2583–99. 25947369 10.1007/s11064-015-1581-6 PMC4636982

[6] Li X Ruan C Zibrila AI. Children with autism spectrum disorder present glymphatic system dysfunction evidenced by diffusion tensor imaging along the perivascular space. Medicine (Baltim). 2022;48:1–6. 10.1097/MD.0000000000032061 PMC9726346 36482590

[7] Nakada T Kwee IL Igarashi H. Aquaporin-4 functionality and virchow-robin space water dynamics: physiological model for neurovascular coupling and glymphatic flow. Int J Mol Sci . 2017;18:1798. 28820467 10.3390/ijms18081798 PMC5578185

[8] Patterson JL Carapetian SA Hageman JR. Febrile seizures. Pediatr Ann. 2013;42:249–54. 24295158 10.3928/00904481-20131122-09

[9] Dougherty D Duffner PK Baumann RJ. Febrile seizures: clinical practice guideline for the long-term management of the child with simple febrile seizures. Pediatrics. 2008;121:1281–6. 18519501 10.1542/peds.2008-0939

[10] Crespo M León-Navarro DA Martín M. Glutamatergic system is affected in brain from an hyperthermia-induced seizures rat model. Cell Mol Neurobiol. 2022;42:1501–12. 33492599 10.1007/s10571-021-01041-2 PMC11421758

[11] Ma L McCauley SO. Management of pediatric febrile seizures. J Nurse Pract. 2018;14:74–80.

[12] Salimeen MSA Liu C Li X. Exploring variances of white matter integrity and the glymphatic system in simple febrile seizures and epilepsy. Front Neurol. 2021;12:1–12. 10.3389/fneur.2021.595647 PMC8097149 33967932

[13] Vestergaard M Pedersen CB Sidenius P. The long-term risk of epilepsy after febrile seizures in susceptible subgroups. Am J Epidemiol. 2007;165:911–8. 17267419 10.1093/aje/kwk086

[14] Eun BL Abraham J Mlsna L. Lipopolysaccharide potentiates hyperthermia-induced seizures. Brain Behav. 2015;5:1–10. 26357586 10.1002/brb3.348 PMC4559014

[15] Marchi N Banjara M Janigro D. Blood-brain barrier, bulk flow, and interstitial clearance in epilepsy. J Neurosci Methods. 2016;260:118–24. 26093166 10.1016/j.jneumeth.2015.06.011 PMC4835226

[16] Biedro A Kubik A Kaci M. ScienceDirect Dilatation of Virchow – Robin spaces in children hospitalized at pediatric neurology department. Neurol Neurochir Pol. 2014;8:7–12. 10.1016/j.pjnns.2013.12.002 24636769

[17] Feldman RE Rutland JW Fields MC. Quantification of perivascular spaces at 7 T: a potential MRI biomarker for epilepsy. Seizure. 2018;54:11–8. 29172093 10.1016/j.seizure.2017.11.004 PMC5909959

[18] Gorter JA Van Vliet EA Aronica E. Status epilepticus, blood-brain barrier disruption, inflammation, and epileptogenesis. Epilepsy Behav. 2015;49:13–6. 25958228 10.1016/j.yebeh.2015.04.047

[19] Banerjee G Kim HJ Fox Z. MRI-visible perivascular space location is associated with Alzheimer’s disease independently of amyloid burden. Brain. 2017;140:1107–16. 28335021 10.1093/brain/awx003

[20] Potter GM Chappell FM Morris Z. Cerebral perivascular spaces visible on magnetic resonance imaging: development of a qualitative rating scale and its observer reliability. Cerebrovasc Dis. 2015;2015:224–31. 10.1159/000375153 PMC4386144 25823458

[21] Cai K Tain R Das S. The feasibility of quantitative MRI of perivascular spaces at 7T. J Neurosci Methods. 2015;256:151–6. 26358620 10.1016/j.jneumeth.2015.09.001 PMC4651825

[22] Ramirez J Berezuk C McNeely AA. Visible Virchow-Robin spaces on magnetic resonance imaging of Alzheimer’s disease patients and normal elderly from the Sunnybrook dementia study. J Alzheimer’s Dis. 2015;43:415–24. 25096616 10.3233/JAD-132528

[23] Niazi M Karaman M Das S. Quantitative MRI of perivascular spaces at 3T for early diagnosis of mild cognitive impairment. Am J Neuroradiol. 2018;39:1622–8. 30093484 10.3174/ajnr.A5734 PMC6128735

[24] Liauw L Grond JVD Slooff V. Differentiation between peritrigonal terminal zones and hypoxic-ischemic white matter injury on MRI. Eur J Radiol. 2008;65:395–401. 17537605 10.1016/j.ejrad.2007.04.016

[25] Ballerini L Lovreglio R Valdés Hernández MDC. Perivascular spaces segmentation in brain MRI using optimal 3D filtering. Sci Rep. 2018;8:1–11. 29391404 10.1038/s41598-018-19781-5 PMC5794857

[26] Liu C Habib T Salimeen M. Quantification of visible Virchow–Robin spaces for detecting the functional status of the glymphatic system in children with newly diagnosed idiopathic generalized epilepsy. Seizure. 2020;78:12–7. 32151968 10.1016/j.seizure.2020.02.015

[27] Dubé CM Brewster AL Baram TZ. Febrile seizures: mechanisms and relationship to epilepsy. Brain Dev. 2009;31:366–71. 19232478 10.1016/j.braindev.2008.11.010 PMC2698702

[28] Ha J Choi J Kwon A. Interleukin-4 and tumor necrosis factor-alpha levels in children with febrile seizures. Seizure. 2018;58:156–62. 29729582 10.1016/j.seizure.2018.04.004

[29] Koh S. Role of neuroinflammation in evolution of childhood epilepsy. J Child Neurol. 2018;33:64–72. 29246095 10.1177/0883073817739528

[30] Abbott NJ. Astrocyte – endothelial interactions and blood – brain barrier permeability *. J Anat. 2002;629:638. 10.1046/j.1469-7580.2002.00064.x PMC1570746 12162730

[31] Janigro D. Cerebral waste accumulation and glymphatic clearance as mechanisms of human neurological diseases. J Neurol Neuromedicine. 2016;1:15–9. 30506062 10.29245/2572.942X/2016/7.1082 PMC6261417

[32] Puvenna V Engeler M Banjara M. Is phosphorylated tau unique to chronic traumatic encephalopathy? Phosphorylated tau in epileptic brain and chronic traumatic encephalopathy. Brain Res. 2016;1630:225–40. 26556772 10.1016/j.brainres.2015.11.007 PMC4853900

[33] Iliff JJ Lee H Yu M. Technical advance Brain-wide pathway for waste clearance captured by contrast-enhanced MRI. J Clin Invest. 2013;123:1299–309. 23434588 10.1172/JCI67677 PMC3582150

[34] Girouard H Iadecola C. Neurovascular coupling in the normal brain and in hypertension, stroke, and Alzheimer disease. J Appl Physiol. 2006;100:328–35. 16357086 10.1152/japplphysiol.00966.2005

[35] Nedergaard M. Garbage truck of the brain. Science (80-). 2013;340:1529–30. 10.1126/science.1240514 PMC3749839 23812703

[36] Abbott NJ. Evidence for bulk flow of brain interstitial fluid: Significance for physiology and pathology. Neurochem Int. 2004;45:545–52. 15186921 10.1016/j.neuint.2003.11.006

[37] Iliff JJ Wang M Zeppenfeld DM. Cerebral arterial pulsation drives paravascular CSF-Interstitial fluid exchange in the murine brain. J Neurosci. 2013;33:18190–9. 24227727 10.1523/JNEUROSCI.1592-13.2013 PMC3866416

[38] Sokrab TO Kalimo H Johansson BB. Parenchymal changes related to plasma protein extravasation in experimental seizures. Epilepsia. 1990;31:1–8. 2303007 10.1111/j.1528-1157.1990.tb05352.x

